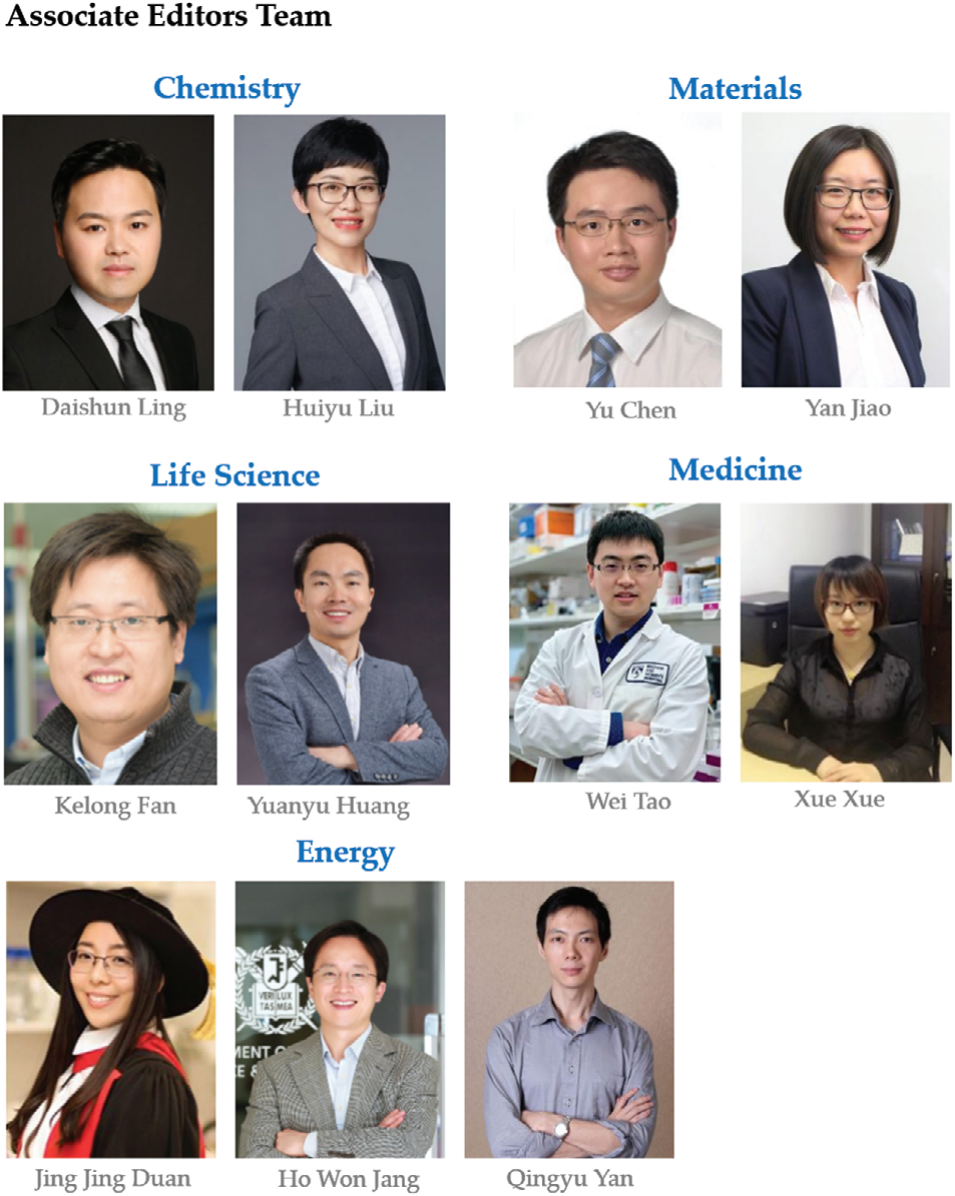# *Exploration*: Explore a better future with advanced science and technology

**DOI:** 10.1002/EXP.20210100

**Published:** 2021-09-01

**Authors:** Yang Liu, Bingyang Shi, Xing‐Jie Liang

**Affiliations:** ^1^ Henan Key Laboratory of Brain Targeted Bio‐nanomedicine School of Pharmacy Henan University Henan China; ^2^ Henan‐Macquarie University Joint Centre for Biomedical Innovation School of Life Sciences Henan University Henan China; ^3^ Department of Biomedical Sciences Faculty of Medicine & Health Sciences Macquarie University Sydney New South Wales Australia; ^4^ Key Laboratory for Biological Effects of Nanomaterials and Nanosafety National Center for Nanoscience and Technology Chinese Academy of Sciences Beijing China

Exploration is the nature of human aspiration to seek innovation through discovery. Ever since the Age of Exploration, which rooted in new technologies and ideas, the massive new discoveries have tremendously accelerated the processes of civilization and scientific development. Some believe that we are emerging into a new theoretical period beyond the Information Age, where creativity and imagination will become the primary creators of economic values, namely the “Imagination Age.” Indeed, traditional industries have already been pressured from the vast advancement of innovative technologies, limiting their vitality. Even the currently booming IT industry is embarking on a certain level of challenge and difficulty in the Post‐Information Age and hankering to prepare for what is to come in the near future. In the present day, the extreme abundance of knowledge demands much more efforts from a person in becoming an expert in different fields. In a sense, the bar of becoming a “human encyclopedia” and to excise interdisciplinary communications has been greatly raised. Scientists and engineers are anxious to have much more in‐depth exchange of ideas from their completely different backgrounds. The same demand echo in the public, which yearns for the excitement of technological singularity, while also concerned about the unforeseeable changes it may lead to. With this issue in mind, it is our mission and vision to provide an easy access to profound communications among science, technology, and public voices.

*Exploration* is an international, cross‐disciplinary and frontline open‐access journal, aiming to promote the development of interdisciplinary sciences and technologies by publishing state‐of‐the‐art short communications, research articles, reviews, perspectives, and commentaries. It focuses on crossing the boundaries of conventional research disciplines, disseminating impactful research to the general public, and inspiring global multidisciplinary collaborations. *Exploration* envisions to provide insights for future science and technology, to catalyze novel integrations of cross‐disciplinary studies, as well as to explore new insights and methods to overcome the obstacles for a sustainable future for society. It welcomes comprehensive articles on cutting‐edge studies utilizing the perspectives and tools on nanoscale. Topics of particular interest include, but are not limited to, biology and biotechnology, medicine and biomedical engineering, chemistry, material sciences, physics and quantum technology, nanoscience and technology, optics, photonics, electronics and robotics, astronomy and planetary sciences, energy science and technology, engineering, mathematics and computer sciences, and any other emerging interdisciplinary research fields.


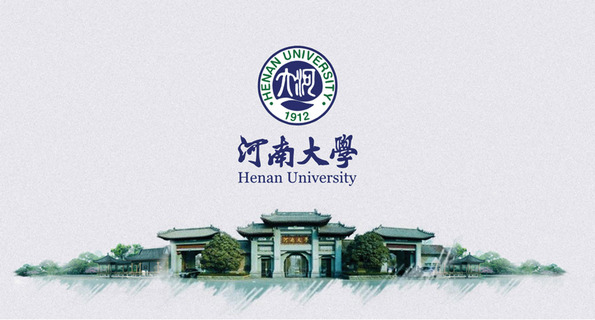


This new journal is co‐published by Wiley, Henan University in China, and the Chinese Association of Nanobiology. The Chinese Association of Nanobiology, encompassed under the Biophysical Society of China, is the leading association in nanotechnology in China with an esteemed reputation and academic influence. Henan University is one of the oldest public and key universities in China, founded back in 1912. At the beginning of its foundation, it was named as the Preparatory School for Further Study in Europe and America, and then changed to the National Henan University and subsequently, restored the name Henan University after the People's Republic of China was founded. The Chinese Ministry of Education named the biology discipline of Henan University in the “Double First‐Class Scheme” in 2017. The three campuses of Henan University locate in both Kaifeng and Zhengzhou. Kaifeng is a famous historic city and was the capital of China during seven different dynasties, including the famous Song dynasty. Zhengzhou is the capital city of Henan province and is one of the National Central Cities in China, the “Zhengzhou metropolitan area” includes Zhengzhou and Kaifeng is the core area of the Central Plains Economic Zone of China.

A group of accomplished scientists from crossing a collection of top‐notch world research groups have teamed up as the gatekeepers for *Exploration*. The Editor‐in‐Chief is Prof. Xing‐Jie Liang from National Center for Nanoscience and Technology, president of Chinese Association of Nanobiology, AIMBE fellow, Faculty Member of F1000 Research. The executive Editor‐in‐Chief is Prof. Bingyang Shi, Honorary Dean of School of Pharmacy, Henan University and Executive Director of Henan‐Macquarie Joint Centre for Biomedical Innovation. Both Editors‐in‐Chief oversee the entire publication process of *Exploration*. We are also proud to introduce our eleven Associate Editors: Prof. Daishun Ling from Shanghai Jiao Tong University (China) and Prof. Huiyu Liu from Beijing University of Chemical Technology (China) are responsible for the chemistry sector. Prof. Jing Jing Duan from Nanjing University of Science and Technology (China), Prof. Ho Won Jang from Seoul National University (Korea), and Prof. Qingyu Yan from Nanyang Technological University (Singapore) are responsible for the energy team. Prof. Kelong Fan from the Institute of Biophysics, CAS, (China) and Prof. Yuanyu Huang from the Beijing Institute of Technology (China) are responsible for the life science team. Prof. Yu Chen from the Shanghai University (China) and Prof. Yan Jiao from the University of Adelaide (Australia) are responsible for the materials sector. Prof. Wei Tao from Harvard Medical School (USA) and Prof. Xue Xue from the Nankai University (China) are responsible for the medicine team. We are also very proud to invite 31 Senior Editorial Board Members and 29 Editorial Board Members from the United States, Australia, Singapore, Sweden, Japan, Ireland, Korea, Germany, and China, which include almost every major research field and cross‐disciplinary field. We firmly believe that the diverse insights and expertise offered by this elite editorial team will enable our authors to present the most crucial, insightful, and cutting‐edge research achievements, and provide both our authors and reviewers with a highly efficient peer‐review and postacceptance process. It is our confident belief that, with you as our passionate readers, resourceful authors, critical commentators, and reviewers, *Exploration* will evolve into a leading open access journal in the cross‐field.


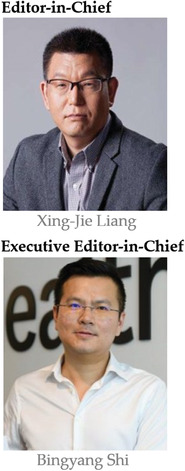


As a final note, the word “Exploration” is suggested to be originated from a hunters' term meaning “to scout the hunting area for game by shouting.” Let us start the hunting for fascinating science and a better future with our *Exploration* team.